# Changes in plasma EBV-DNA and immune status in patients with nasopharyngeal carcinoma after treatment with intensity-modulated radiotherapy

**DOI:** 10.1186/s13000-019-0798-0

**Published:** 2019-03-14

**Authors:** Qi Chen, Wei Hu, Huacai Xiong, Shenpeng Ying, Yanyun Ruan, Bo Wu, Hongsheng Lu

**Affiliations:** 1 Precision Medicine Center, Taizhou Central Hospital (Taizhou University Hospital), Taizhou, Zhejiang People’s Republic of China; 2 Department of Radiotherapy, Taizhou Central Hospital (Taizhou University Hospital), Taizhou, Zhejiang 318000 People’s Republic of China; 3 Department of Pathology, Taizhou Central Hospital (Taizhou University Hospital), Taizhou, Zhejiang People’s Republic of China

**Keywords:** Plasma EBV-DNA, Intensity-modulated radiotherapy, Nasopharyngeal carcinoma, PD-1, Treg cells

## Abstract

**Background:**

Previous studies reported the early diagnostic values of plasma Epstein-Barr virus (EBV)-DNA. The present study aimed to assess the relationship between the concentration of plasma EBV-DNA and the number of CD8^+^PD-1^+^(programmed cell death-1,PD-1) and regulatory T (Treg) cells in patients with nasopharyngeal carcinoma (NPC) who were treated with intensity-modulated radiotherapy (IMRT).

**Methods:**

This study included 37 patients treated with IMRT. Peripheral blood samples were collected two times for each patient, before radiation therapy and 1 week after the treatment. Further, the numbers of CD4^+^, Treg, CD8^+^, and CD8^+^PD1^+^ cells were determined by flow cytometry.

**Results:**

The changes after IMRT were determined by comparing the numbers of neutrophils, lymphocytes, CD4^+^, Treg, CD8^+^, CD8^+^PD1^+^ cells, and the concentration of plasma EBV-DNA between pretreatment and post-treatment groups. IMRT could reduce the expression level of PD-1 and the number of Treg cells. The concentration of plasma EBV-DNA and the expression level of CD8^+^PD-1^+^ were closely associated with the occurrence and development of NPC. Thus, EBV-DNA can be used as an important marker for early diagnosis, and IMRT can strongly reduce the copies of EBV-DNA.

**Conclusions:**

This study showed that IMRT could reverse T-cell exhaustion and reduce the copies of EBV-DNA. In clinical practice, plasma EBV-DNA is a sensitive biomarker for diagnosis, prognosis, and evaluation of clinical efficacy.

## Introduction

Nasopharyngeal carcinoma (NPC) is one of the head and neck epithelial cancers and is a serious threat to human health. According to the latest statistics, about 80% of patients with NPC were observed in Asia, especially in Southeast Asia and south China. Based on the previous reports, the estimated incidence rate of NPC in China was 60.6 per 100,000, and the mortality rate was 34.1 per 100,000 [1].

With the development of radiotherapy techniques in recent years, intensity-modulated radiotherapy (IMRT) has been widely applied for treating patients with NPC. Also, the prognosis of early-stage NPC has been significantly improved. However, with the development of tumor treatment, a number of studies have discovered the immunogenicity of radiotherapy [2–4]. Epstein-Barr virus (EBV) causes several lymphomas and hence has been considered to cause NPC. Plasma EBV-DNA is regarded as a significant biomarker for NPC. Circulating cancer-derived EBV-DNA in plasma has been shown to be associated with the early screening of patients with NPC [5].

PD-1, a new indispensable member of CD28/B7 co-stimulating signals, is a surface inhibitory receptor having critical functions in regulating T-cell activation and tolerance [6]. It is also an important marker of T-cell exhaustion.

The present study aimed to determine the relationship between the concentration of plasma EBV-DNA and the numbers of CD8^+^PD-1^+^ and regulatory T (Treg) cells in patients with NPC who were treated with IMRT.

## Materials and methods

### Study population

This was an observational study in which data were collected between July 2015 and June 2016. The criteria used in the study were according to the guidelines for diagnosis, treatment, and follow-up of NPC. A total of 40 patients with NPC were selected from Taizhou Central Hospital (Zhejiang, China). Of these, 37 patients were treated with IMRT, and the others lost the follow-up because they moved to another hospital for treatment. All patients underwent primary screening before the treatment. The basic information of these patients was collected, including age, gender, clinical stage, pathological classification, treatment regimen, and follow-up records. Patients were restaged according to the seventh edition of the American Joint Committee on Cancer tumor, node, metastasis (TNM) staging system released in 2010. Besides, 40 healthy individuals were included as controls. The demographic and clinical characteristics of the patients are summarized in Table 1. No significant difference was found in sexuality and age between patients with NPC and controls (*P* > 0.05). The clinical stages were divided into early stage and late stage (early stage = I + II; late stage = III + IV).

**Table 1:** Baseline characteristics of the 37 patients and controls

Clinical parameters	NPC patients (*N* = 37)	Healthy Donors (*N* = 40)	
Age (years)			
Median (range)	53 (37–90)	50 (20–73)	*p* = 0.072
	> = 53 19	> = 50 22	
	< 53 18	< 50 18	
Gender			
Male	25	23	*p* = 0.362
Female	12	17	
Clinical stage			
I + II (early stage)	16		
III + IV (late stage)	21		
Tumor stage^a^			
T1 + T2	23		
T3 + T4	14		
Lymph node stage^a^			
N0	5		
N1 + N2 + N3	32		
Metastasis stage^a^			
Mo	34		
M1	3		
Tumor differentiation			
Keratinizing	3		
Non-Keratinizing	34		

*NPC* nasopharyngeal carcinoma

^a^According to the AJCC tumor-node-metastases (TNM) staging system, 2010

### Ethics statement

This study was approved by the Ethics Committee of Taizhou Central Hospital. All patients signed the informed consent form before the samples were collected.

### Samples

The peripheral blood samples (3 mL) were collected from patients with NPC before treatment (Pre) and after treatment (Post), and from healthy donors (HD). Anticoagulant samples were used for flow cytometry analysis. Plasma samples were centrifuged for 5 min at 800 rpm and tested by plasma EBV-DNA assay.

### Monoclonal antibodies

Fluorochrome-conjugated monoclonal antibodies (mAbs) were CD4-FITC (Fluoresceine isothiocyanate) clone SK3, CD25-APC (Allophycocyanin) clone 2A3, CD127-PERCP (Peridinin chlorophyll protein)-CY5.5 clone HIL-7R-M21, mouse-IgG1-FITC clone X40, mouse-IgG-PE (Phycoerythrin) clone MOPC-21, mouse-IgG1-APC clone SJ25C1, mouse-IgG1- PERCP-CY5.5 clone X40, CD4- PERCP-CY5.5 clone SK3, CD8-FITC clone 2D1, and CD279(PD-1)-PE clone EH12.1 (BD Biosciences, CA, USA).

### Flow cytometry analysis

**Fig. 1:**
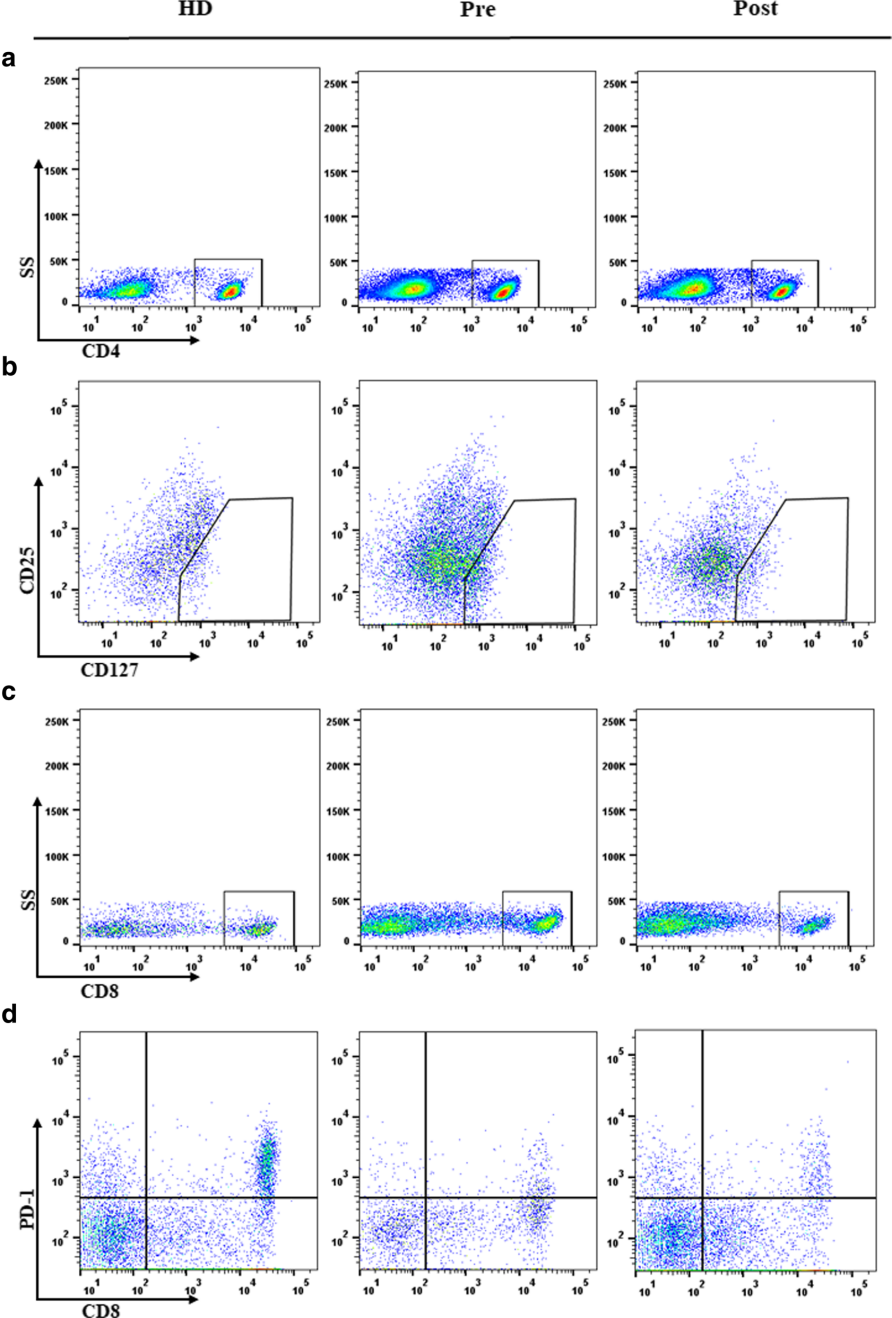
Flow cytometry analysis of the numbers of CD4^+^, CD8^+^ T, CD8^+^ PD-1^+^ T, and Treg cells. Abbreviations: **a** Number of CD4^+^ T cells; **b** number of Treg cells; **c** number of CD8^+^ T cells; **d** number of CD8^+^PD-1^+^T cells; HD, health honor; Pre, pre-radiotherapy; Post, post-radiotherapy

Flow cytometry analysis was performed to determine the numbers of neutrophils, lymphocytes, CD4^+^, Treg , CD8^+^, and CD8^+^PD1^+^ (Fig. 1). Peripheral blood mononuclear cells were washed with phosphate-buffered saline (PBS) with 5% heparin-activated fetal calf serum and then stained for the surface markers CD4 (FITC), CD25 (APC), and CD127 (PERCP-CY5.5)) according to the manufacturer’s instructions. Another tube was added with CD8^+^-FITC/PD-1^+^-PE/CD4^+^- PERCP-CY5.5. The cells were washed twice with PBS and analyzed immediately using BD-FACS AriaII cytometer (BD Biosciences, CA, USA) and FlowJo software (BD Biosciences). Quadrants and box gates were set about isotype controls, and the percentages of the Treg and CD8^+^ PD-1^+^ subsets were accordingly calculated.

### Use of hemocytometer

Routine blood specimens were anticoagulated with K2-EDTA (Ethylene Diamine Tetraacetic Acid). Sysmex XE-2100 (Sysmex Corp., Kobe, Japan) and reagents were used to obtain the total numbers leukocytes and lymphocytes.

### DNA extraction and plasma EBV-DNA assay

Polymerase chain reaction (PCR) was used to detect the concentration of plasma EBV-DNA. The plasma samples were stored at − 80 °C for EBV-DNA assay. DNA was extracted from plasma. The concentration of EBV-DNA was measured by quantitative reverse transcription PCR (RT-qPCR).

### Clinical treatment

Peripheral blood samples were collected two times for each patient before radiation therapy and 1 week after the treatment.

### Statistical analysis

In the present study, SPSS 17.0 software (IBM, NY, USA) was used to analyze the data, and GraphPad Prism 6 (GraphPad Software, Inc., CA, USA) was used to generate graphs. Shapiro–Wilk test was used to test the normality of our data. As the data became skewed, the mean could not provide the best central location for the data because the skewed data dragged it away from the typical value. However, the median best retained its position and was not as strongly influenced by the skewed values. A paired *t* test was used to compare the indexes between the pretreatment and post-treatment groups when the data were normally distributed; otherwise, the Wilcoxon signed-rank test was applied. The Mann–Whitney *U* test was used to compare the samples between pretreatment and HD. The receiver operating characteristic (ROC) curve was used to determine the sensitivity and specificity. The chi-square test was applied to compare categorical data between the groups. One-way analysis of variance was used to compare the indexes of patients with NPC at different clinical stages. Least significant difference test was performed between every two stages. The results were considered statistically significant when the *P* values were less than 0.05.

## Results

### Description of demographic characteristics

This study included 37 patients diagnosed with NPC having no chronic diseases, such as autoimmune diseases and other inflammatory diseases. Of these, 25 were men and 12 were women. The clinical characteristics of the patients before treatment are listed in Table 1. In addition, the distributions of patients with NPC according to the TNM classification system are also presented in Table 1. Based on the last follow-up, five patients had died.

### Number of various immune cells and concentration of plasma EBV-DNA in patients with NPC before and after treatment with IMRT and HD

The changes in HD were compared, and the changes in different stages were also investigated.

Further, the indexes were compared and analyzed between the pretreatment and healthy donor groups. The number of immune cells and the concentration of EBV-DNA obviously increased in the pretreatment group [*P* < 0.01, odds ratio (OR) 0.02, 95% confidence interval (CI) 0.00–0.20)]. No significant difference was observed in the number of Treg cells between the pretreatment and healthy donor groups, although the *P* value was higher than 0.05 (OR 0.86, 95% CI 0.35–2.10). The numbers of lymphocytes and CD4^+^, CD8^+^, and CD8^+^PD-1^+^ cells obviously decreased in the pretreatment group (*P* < 0.05, OR 9.50, 20.06, and 3.17) (Table 2). Importantly, the study focused on the number of lymphocytes, neutrophil–lymphocyte ratio (NLR), concentration of plasma EBV-DNA, and numbers of CD4^+^, CD8^+^, and CD8^+^PD-1^+^ cells to plot ROC curves. The concentration of plasma EBV-DNA had the highest values of area under the curve, sensitivity, and specificity (Table 3).

**Table 2:** Pre- and post-radiotherapy findings of studied patients compared with healthy levels

Parameters	HD	Pre	Post	*P* 1	*P* 2	OR	95%CI
N (10^6^/ml)	3.30 (2.00–5.90)	3.90 (1.70–13.20)	3.31 (0.95–23.49)	0.06	0.03	0.46	(0.18, 1.15)
L (10^6^/ml)	2.05 (1.00–3.50)	1.30 (0.20–6.00)	0.43 (0.12–2.32)	0.00	0.00	29.71	(3.68, 239.94)
NLR (10^6^/ml)	1.60 (0.90–3.30)	2.47 (1.29–29.50)	8.24 (1.34–68.21)	0.00	0.00	0.19	(0.06, 0.55)
EBV DNA (copies/ml)	56.11 (0.00–3213.50)	1132.56 (0.00–19065.22)	35.67 (0.00–5678.50)	0.00	0.00	0.02	(0.00, 0.20)
CD4^+^ (10^6^/ml)	0.70 (0.18–1.20)	0.37 (0.05–1.09)	0.07 (0.00–073)	0.00	0.00	9.50	(2.81, 32.10)
Treg (10^6^/ml)	0.08 (0.04–0.23)	0.09 (0.01–0.26)	0.02 (0.00–0.16)	0.68	0.00	0.86	(0.35, 2.10)
CD8^+^ (10^6^/ml)	8.49 (3.02–14.85)	3.42 (0.00–7.16)	0.22 (0.00–0.75)	0.00	0.00	20.06	(4.21, 95.56)
CD8^+^PD1^+^ (10^6^/ml)	0.19 (0.05–0.45)	0.13 (0.03–0.58)	0.05 (0.01–0.19)	0.02	0.00	3.17	(1.21, 8.30)

*N* absolute neutrophil count, *L* absolute lymphocyte count, *NLR* neutrophil lymphocyte ratio, *Treg* CD4^+^CD25^+^CD127^low^, *P1* Healthy Donor& Pre-radiotherapy, *P2* Pre-radiotherapy &Post-radiotherapy, *OR* the odds ratio, *95%CI* 95% confidence intervals

**Table 3:** ROC analysis for EBV DNA, Treg and CD8^+^PD-1^+^

Parameters	AUC	*p*	Sensitivity	Specificity
L	0.796	0.000	0.027	1
NLR	0.775	0.000	0.892	0.525
EBV DNA	0.912	0,000	0.838	0.925
CD4^+^	0.770	0.000	0	1
CD8^+^	0.947	0.000	0	1
CD8^+^PD-1^+^	0.650	0.024	0.054	0.975

*AUC* Area Under ROC Curve

**Fig. 2:**
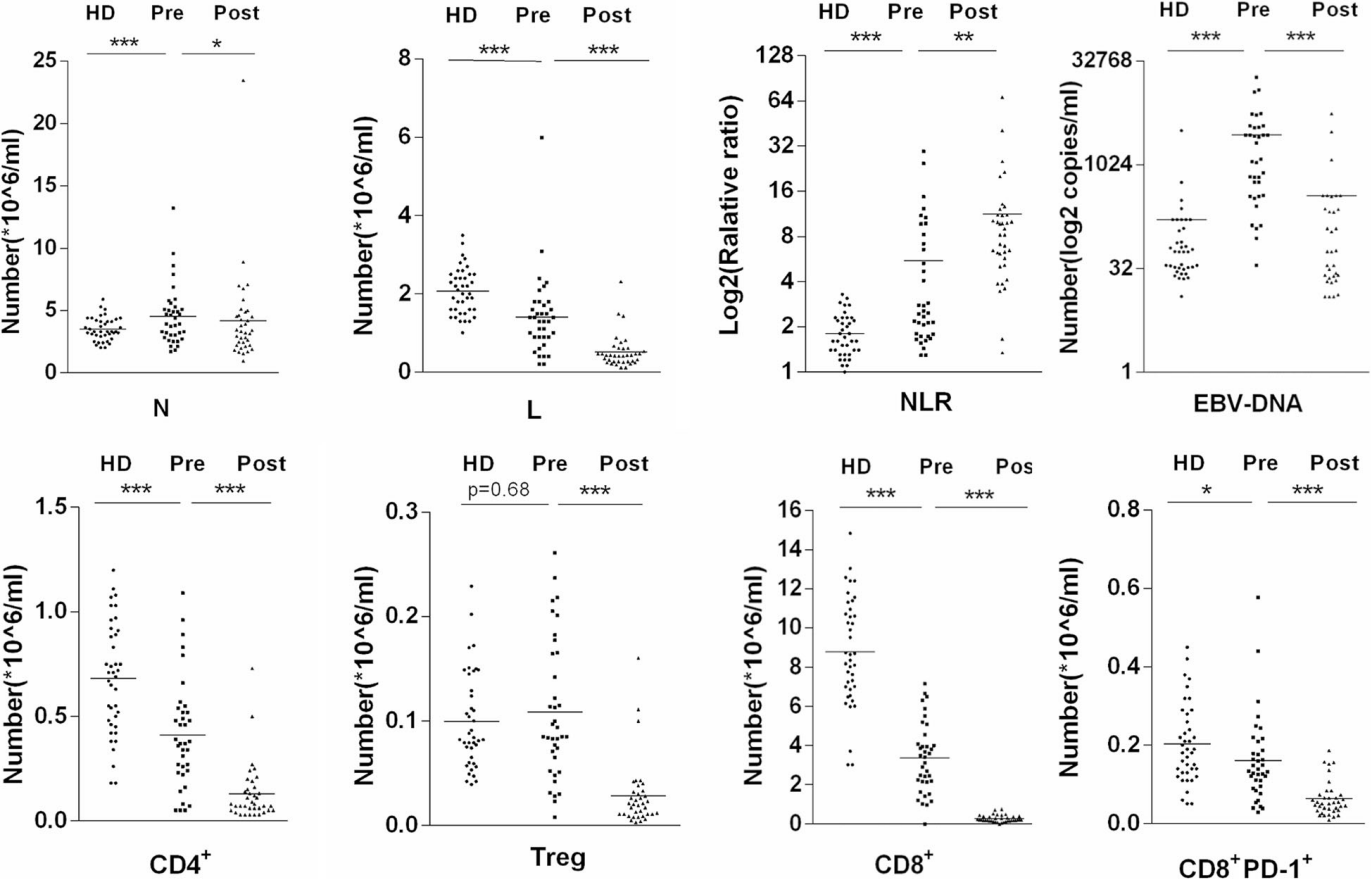
Pre- and post-radiotherapy findings of patients compared with healthy donors (*P < 0.05, **P < 0.01, ***P < 0.001). Abbreviations: HD, healthy donors; L, absolute lymphocyte count; N, absolute neutrophil count; NLR, neutrophil–lymphocyte ratio Pre, pre-radiotherapy; Post, post-radiotherapy; Treg, CD4^+^CD25^+^CD127^low^

**Fig. 3:**
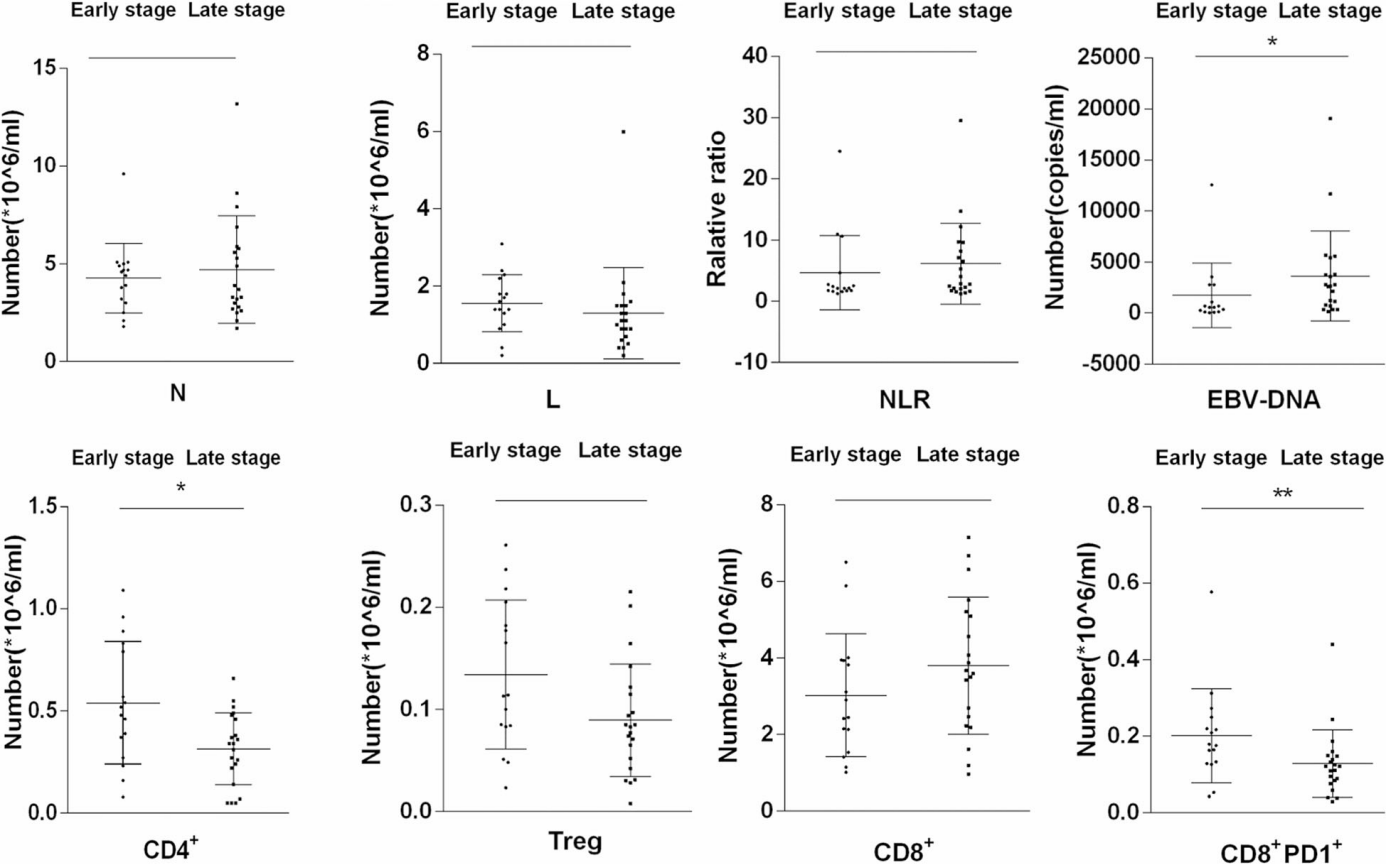
Expression levels of indexes in patients with NPC at different clinical stages (*P < 0.05, **P < 0.01). Abbreviations: early stage, I + II; late stage, III + IV

**Fig. 4:**
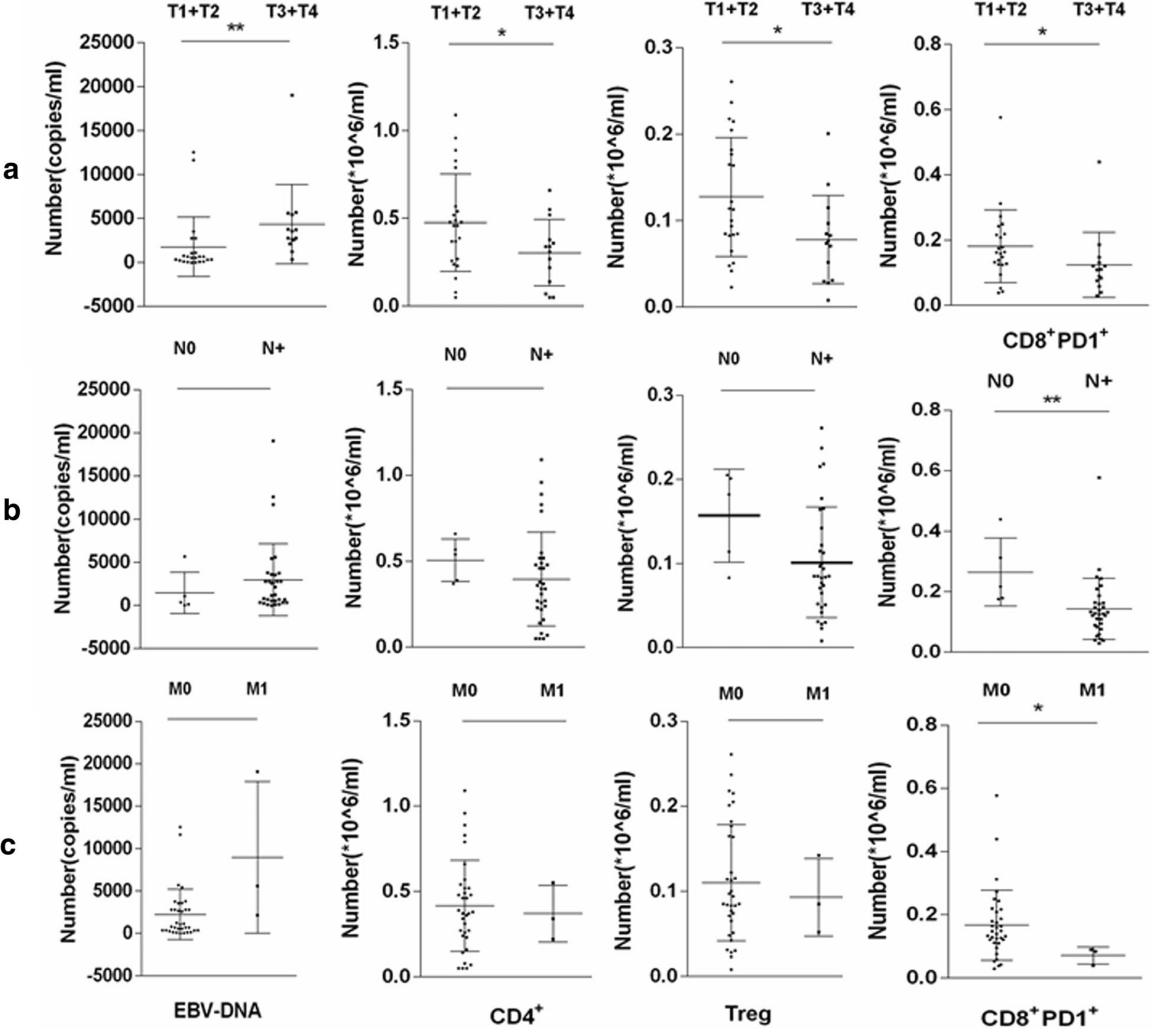
Expression levels of indexes in NPC patients with different TNM stages (*, *P* < 0.05, **,*P* < 0.01). Abbreviations: **a** Comparison of EBV DNA, CD4^+^ T cells, Treg, CD8^+^ PD-1^+^ T cells in different T stages; **b** Comparison of EBV DNA, CD4^+^ T cells, Treg, CD8^+^ PD-1^+^ T cells in different N stages; **c** Comparison of EBV DNA, CD4^+^ T cells, Treg, CD8^+^ PD-1^+^ T cells in different M stages

The results were also compared between the pretreatment and post-treatment groups. The number of lymphocytes, NLR, concentration of plasma EBV-DNA, and numbers of CD4^+^, CD8^+^, and CD8^+^PD-1^+^ cells remarkably decreased after treatment with IMRT (*P* < 0.01) (Fig. 2). In the present study, the absolute numbers of neutrophils and lymphocytes, neutrophil–lymphocyte ratio, and numbers of CD4^+^ T, CD8^+^ T, CD8^+^ PD-1^+^, and Treg cells. In 37 patients with NPC, different clinical stages showed different expression levels. The late stage showed a significantly higher concentration of plasma EBV-DNA compared with other stages (*P* < 0.05). At the same time, the late stage showed a notably fewer number of CD4^+^ T cells (*P* < 0.05) and CD8^+^ PD-1^+^ T cells compared with other stages (*P* < 0.01) (Fig. 3). The study also analyzed the relationship between TNM stages and various indexes (Fig. 4). The stage of T3 + T4 showed a significantly higher concentration of plasma EBV-DNA (*P* < 0.01). Conversely, other indexes showed lower values (*P* < 0.05). Lymphatic and distant metastases had fewer numbers of CD8^+^ PD-1^+^ T cells compared with other stages (*P* < 0.05).

### Relationship between the prognosis and immune status for patients with NPC

**Fig. 5:**
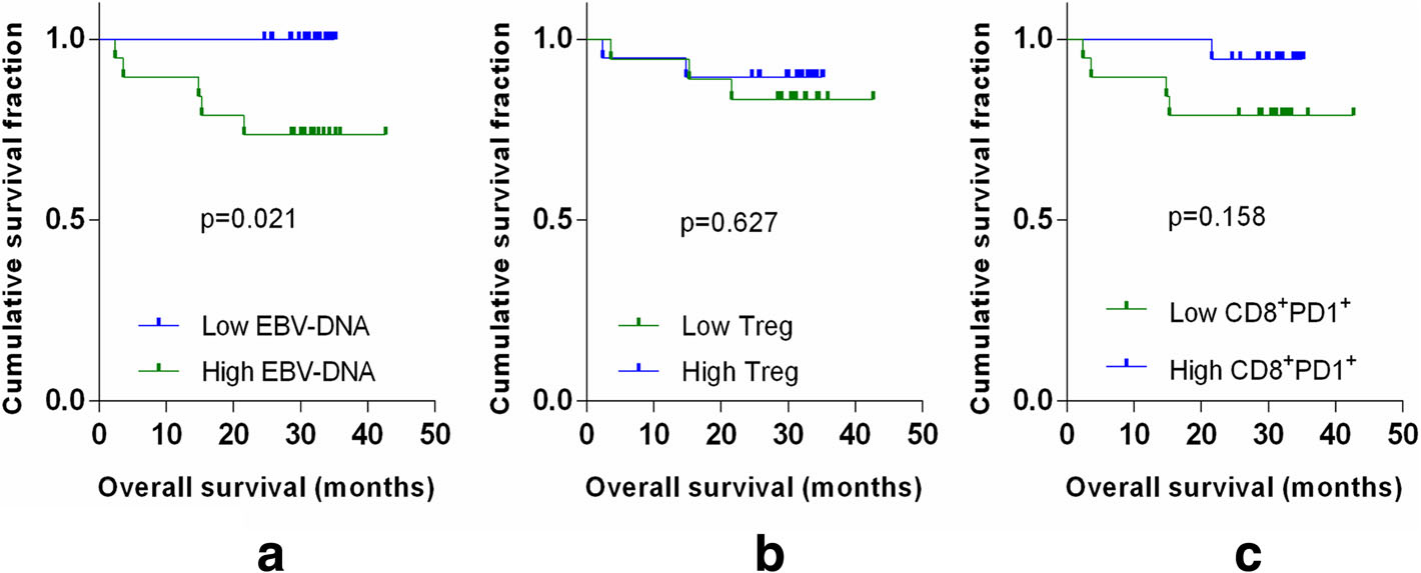
Kaplan–Meier analysis of OS. **a** The concentration of plasma EBV-DNA was evaluated according to the median (1132.56 copies/mL); more than the median was high, while the opposite was low. **b** The number of Treg cells was evaluated according to the median (0.085 × 10^6^); more than the median was high, while the opposite was low. **c** The number of CD8^+^ PD-1^+^ T cells was evaluated according to the median (0.133 × 10^6^); more than the median was high, while the opposite was low

All patients were followed up. Kaplan–Meier survival analysis showed that the concentration of plasma EBV-DNA was positively associated with worse overall survival (OS) (*P* = 0.021). The numbers Treg and CD8^+^ PD-1^+^ T cells were negatively associated with worse OS (*P* = 0.627, *P* = 0.158) (Fig. 5).

## Discussion

NPC is a kind of malignant tumor originating from the epithelial tissue. In China, it is mainly distributed in southern provinces, such as Guangdong, Guangxi, Fujian, Zhejiang, and so on. Despite a traditional treatment that combines radiotherapy with chemotherapy, NPC has a high mortality rate because of tumor metastasis [6–8]. Therefore, novel modes of detecting and treating NPC need to be developed and validated [7, 8]. Recently, IMRT has been widely used for treating NPC. Novel molecular markers that can help detect the risk of tumor recurrence are urgently required [9].

Chen et al. [10] found that the number of Treg cells obviously increased in patients with NPC. In the present study, the number of Treg cells increased in the pretreatment group compared with the healthy donor group, though with some insignificant differences. Also, the number of Treg cells significantly decreased after IMRT. Treg cells control autoimmune reactivity of the T-cell subgroup. They are a key factor in tumor immune escape [11]. The number of Treg cells, an immunosuppressive index, increased in patients with NPC, and IMRT treatment could reduce immunosuppression. The difference in the number of Treg cells between the present study and previous studies might be due to the sample size and the number of patients.

T lymphocytes are an important part of cellular immunity in vivo. Especially CD8^+^T lymphocytes (CTLs) have a key role in the recognition and killing of tumor cells. First, the activation of T cells requires the recognition of T cell receptor (TCR) TCR and the presentation of APC. Second, the participation of costimulatory signals is also crucial. These two factors synergistically lead to the activation of T cells. The costimulatory molecules are divided into two categories: positive and negative, of which CTLA-4 and PD-1 are negative costimulatory molecules. Compared with CTLA-4, PD-1 regulates immune responses more negatively and widely [12]. Besides, CD8^+^PD-1^+^ cells are considered as a representative subpopulation of negative activation of T cells, and PD-1 inhibits Akt phosphorylation by interfering with CD28-mediated PI3K activation, thereby weakening the activation of T cells by TCR/CD28 signaling [13]. Second, PD-1 reduces the phosphorylation level of PKC, attenuating the effects of PKC and Ras/MAPK. This leads to a decrease in the expression of transcription factors, such as nuclear factor-kappa B and AP-1. PD-1 can inhibit T-cell proliferation, cytokine secretion, and other immune activities [14]. The PD-1 signaling pathway also inhibits the proliferation of T cells by inhibiting the expression of anti-apoptotic gene BCL-XL [15]. Therefore, the CD8^+^PD-1^+^ T-cell subset is an important indicator for T-cell exhaustion.

Some studies reported the expression level of PD-1 in tumors, including NPC, hepatic-cellular cancer, breast cancer, renal cell carcinoma, non-small cell lung carcinoma (NSCLC), high-grade serous carcinoma, and so on [16–20]. Previous studies suggested that PD-1 gene correlated with a poor tumor prognosis, and PD1/ PD-L1 axis had great potential as a treatment target. However, the expression level of PD-1 in NPC is not consistent with that reported in the present study. Larbcharoensub et al. [21] reported that approximately 70% of patients with EBV-positive NPC expressed PD-L1, but this did not correlate with patient’s survival or clinicopathological features. PD-1 in tumors was expressed in only 13 (out of 114) patients. Tang *et al.* assessed the expression level of PD-1 in a cohort of 96 paraffin-embedded NPC samples and confirmed that PD-1 was co-expressed in infiltrating lymphocytes in patients with NPC [22].

Zhang et al. [23] further reported that PD-1 association with PD-L1 in patients with NPC correlated with the worst prognosis of disease-free survival. Chan et al. [24] implied EBV-DNA in plasma specimens of participants who did not have any symptoms of NPC. Fang et al. [16] also reported that blocking of PD-1/PD-L1 checkpoints might be a promising therapeutic approach for patients with EBV-positive NPC.

In this study, the concentration of plasma EBV-DNA and the numbers of Treg and CD8^+^PD-1^+^ cells were closely associated with the occurrence and development of NPC. Because of high sensitivity and specificity, plasma EBV-DNA can be used as an important marker for early diagnosis. To explore the changes after IMRT, the numbers of neutrophils, lymphocytes, and CD4^+^, Treg, CD8^+^, and CD8^+^PD1^+^ cells and the concentration of plasma EBV-DNA, were compared between the pretreatment and post-treatment groups, revealing no significant differences. IMRT can reduce the expression level of PD-1 and Treg cells, suggesting that IMRT can reverse T-cell exhaustion [25, 26]. The concentration of plasma EBV-DNA also notably declined after IMRT. It was assumed that T-cell exhaustion led to a massive amplification of plasma EBV-DNA in patients with NPC.

## Conclusions

This study found that IMRT could reverse T-cell exhaustion and reduce the concentration of EBV-DNA. In clinical practice, plasma EBV-DNA is a sensitive biomarker for diagnosis, prognosis, and evaluation of clinical efficacy. This is also in agreement with previous findings [23, 24].
